# Diseases of the Pancreas

**Published:** 1876-11

**Authors:** J. E. Lockbridge

**Affiliations:** Indianapolis


### Selections.

### DISEASES OF THE PANCREAS.

By J. E. LOCKBRIDGE, Indianapolis.

Heretofore most of the systematic writers on the practice of medicine have treated very slightly the diseases of the pancreas. Some, like Burlow, have ignored them entirely; the classical Sir Thomas Watson, rather than "slight" this organ, devotes just eleven lines to its diseases, but winds up by saying, "I do not know of any remedies" for these complaints. Flint and Wood each gives about one and a half pages to their consideration. It is not my purpose to write a treatise on these complaints, but rather to enter a mild protest against two propositions that all of these writers seem to lay down; these are, that diseases of the pancreas are very rare, and when they do exist the diagnosis is most difficult, if not impossible.

In considering the first of these propositions, I will say that I can see no good reason why we might not expect to encounter diseases of the pancreas as well as almost any other of the adjuvant organs of digestion; and that, too, both primarily and secondarily. Anatomically, it resembles the salivary glands, and in physical properties its secretion is almost identical. The organ derives its blood supply wholly from the hepatic, splenic, and superior mesenteric arteries, and hence trouble at these fountains would be likely to affect it secondarily. In the last ten years of my experience it has been my lot to encounter disease of the organ, in some form, quite frequently; but during the first ten years of my experience I met with not a single case to my present knowledge, because, having been taught that they were "very rare," I doubtless overlooked many cases. Sir Thomas Watson writes that "perhaps he has seen eight or nine cases of cancer of the head of the organ in his experience." I, with not one tithe of the experience or skill that he possesses, have seen quite that number of either cancer, simple hypertrophy, or chronic inflammation of this organ. I am forced to believe that many cases have run their course, either to a favorable or fatal termination, without their real nature ever having been suspected. From having been called in consultation in these cases, and by having them come under my care after having passed through the hands of other physicians, I have learned that they are most apt to be mistaken for the following complaints, taking them in the order in which I here place them: dyspepsia, so-called "liver disease," scirrhus of the pylorus, and hypertrophy of the greater curvature of the stomach, aneurism of the abdominal aorta, enlarged spleen, floating kidney (left), and an accumulation of fæces in the transverse colon from obstruction. This brings us to the attempted refutation of the second proposition, that the diagnosis is next to impossible.

In the outset it would be well to enumerate a set of symptoms that generally accompany affections of this organ; but, by way of parenthesis, I might as well say that there are only two symptoms that are pathognomonic of pancreatic diseases, and these are a tumor situated between the pit of the stomach and umbilicus, and which has been ascertained by elimination to be in the pancreas; and the other symptom is the constant passage through the bowels of the undigested fatty portion of the food; for it is the known function of the pancreatic juice to emulsify fats, and render them suitable for imbibation by the lacteals. But there are scores of other symptoms, some of which I will enumerate: a fixed pain, or rather soreness on pressure over the epigastrium, a disposition to lie on the right side, because the dorsal *decubitus* is rendered painful by the weight of the stomach; and on the left side by that of the liver; the patient is generally affected with a train of gastric or dyspeptic symptoms, such as pyrosis, gastrodynia, nausea, and vomiting, which for the most part come on a few hours after eating; there is often inordinate action of the heart, without alteration in structure or rythm; there is, on very close examination, a deep-seated tumor just above the umbilicus, fixed and roundish, hard and somewhat nodulated in scirrhus, but rather oblong and elastic in simple hypertrophy; there is jaundice, or at least an approach thereto, from pressure on the common duct; there is generally aortic pulsation, and sometimes a *bruit*. As the disease, or diseases, advances, there is emaciation and general malaise; the bowels are either constipated or loose, as the case may be; there is generally an absence of fever, and the tongue is generally coated with a white fur denoting feeble digestion, and the appetite is generally moderately good.

I will now attempt to lay down some rules for the differential diagnosis of the diseases of the organs in question, from those of some of the other organs and parts of this anatomical jumble, with which they have often been confounded. In the case of some of them it is comparatively plain sailing, but in others, I am willing to admit, that it is as difficult as possible. But in none do I consider it impossible, if we take ample time, and bring to bear on the case all of a reasonably good anatomical and physiological knowledge.

As previously indicated, the superficial observer is almost sure to mistake disease of the pancreas for dyspepsia. He encounters a case with the usual symptoms of dyspepsia, and either for a want of tact in detecting anatomical changes, or much oftener from a failure to examine the region *at all* for these changes, he goes to work and plies his remedies with vigor, and directs his treatment altogether to the stomach. He never suspects that he has an hypertrophy, chronic inflammation, or scirrhus of the pancreas, to deal with. After six months or a year, more or less, the patient may *by chance* get well, or his physician may send him off to some mineral spring which happens to possess powerful deobstruent properties, and by this means a cure may be effected. But more often he goes from doctor to doctor, if he does not go to the graveyard in the meantime, until at last he meets with one who detects the true nature of his case, and institutes the proper treatment.

Here is a case in question. Early in June of the present year, and just before I left Virginia, a gentleman came to consult me from a neighboring county. He told me that he had enjoyed almost uninterrupted health until the first day of last January, at which time he took a bad cold, which almost ran into pneumonia before he got over it; and during this time, and for some weeks afterwards, the glands ot his neck were very much swollen. After using iodine on them for some time the swelling entirely disappeared, but still he was not well. When I saw him he was complaining of soreness in the region of his stomach; his appetite was quite good, but yet he had lost forty pounds in flesh, and was still losing; he complained of considerable uneasiness after meals, yet he seemed to digest his food very well; he had rather an inordinate desire for fat meat, but there was no evidence that the fat passed through undigested, or at least he had not observed anything of the kind; there was inordinate action of the heart, the pulse counting one hundred and twenty to the minute, yet the heart was perfectly sound in structure and rhythm. On examination I at once detected a deep-seated tumor between the pit of the stomach and umbilicus; the tumor was pulsating and attended with a faint *bruit;* it was rather oblong in shape, somewhat elastic, and was really less painful under pressure than a line or circle of soreness which surrounded the tumor, and which was caused by its encroachment on neighboring parts or organs. Now for the differentiation of this tumor. It was not in the stomach, for there were really no symptoms of indigestion that would imply that there was a large portion of the stomach involved in organic disease, for the tumor was at least three times the normal size of the pancreas; there was no pyrosis, or water-brash, but rather a sensation of encroachment. It could not be in the liver, for whilst there was rather a jaundiced hue of the skin, which was caused by the pressure on the common bile duct, yet the tumor was in the median line, and the liver was felt in its normal position, and without tenderness or alteration in size. Of course the left kidney and spleen were out of the question; for a floating kidney—two cases of which I have seen, both on the right side, and both in a female—is quite easily made out from its shape and smoothness, and want of tenderness on pressure; and the median position and circumscribed character of the tumor distinguished it from an ague-cake.

We come now to the consideration of this, and other such cases, as regards their discrimination from aneurism of the abdominal aorta at the most common seat, where it is weakened by giving off the cœliac axis; this, perhaps, is the most difficult in most cases to determine. Both tumors are deep-seated; in both there is generally pulsation; in both there is commonly a b*ruit* of some kind. The pancreatic tumor is generally longest transversely, whilst the aneurismal is most often fusiform, and is therefore vertical; the practiced ear will generally distinguish the simple *bruit de souffle* of the pancreatic tumor, caused by its pressure on the aorta, from the well-known aneurismal purr. If the fingers of both hands be gently but persistently insinuated down on either side of the tumor, if it be a pancreatic tumor, it can be raised from its contact with the aorta and the pulsation will cease, as well as the bruit; but in the case of an aneurism the tumor is more fixed, and the pulsation will continue laterally, as well as vertically. Still further, if the patient be placed in the genu-manual or "all-fours" position, the diseased gland will gravitate from the aorta, and the *bruit* will cease unless it be an aneurism.

In my preceding remarks I have been dealing, for the most part, in negations and eliminations; but now let us inquire into the purport of some of the positive signs of pancreatic diseases. The jaundice and gastric symptoms are produced in part by direct pressure; but in many cases I am disposed to attribute the origin of these symptoms to the direct pressure of the tumor on the semilunar ganglion and solar plexus of the sympathetic nerve, with which both an enlarged pancreas and aortic aneurism would come in contact; and I have seen both these maladies produce precisely analogous symptoms on each of these neighboring organs. It is very easy to explain this nervous catenation through the irradiating fibres of the solar plexus and its connection with the pneumogastric. So great was the tumult of the heart in the case of the gentleman, whose case I have been carrying along with me in these remarks for the sake of differentiating the diagnosis, that he could scarcely believe but that his heart was awfully diseased.

Another symptom of vast importance in the diagnosis, is the inordinate desire for fat meats. This is of easy explanation. The emulsifying process, and consequent absorption of the fatty ingesta by the lacteals, is in abeyance for the want of healthy pancreatic juice, and the economy is simply crying for this element of nutrition, and hence the appetite for the fats. Another point of importance is the very *deeply-seated* location of the tumor, which should of itself almost reduce the case to the diagnosis between it and aneurism, and of this I have spoken very fully, but will have occasion to allude to it again.

But do not let us lose sight of the case which I have taken along as a sort of *vade mecum* in this paper. This gentleman had been treated for four or five months by several very intelligent physicians for dyspepsia, but without any improvement, but on the other hand a progressive loss of flesh and strength. They had not discovered the tumor in the hypogastric region, and the patient informed me that neither of them had even examined or *touched* him in this region. From the fact that the family was evidently a scrofulous one, I diagnosed the case as strumous enlargement of the pancreas; and although, from some indications about the case, I could not give a very favorable opinion as to the result, yet the gentleman seemed relieved, as the cause at least of his ailment was probably discovered, for I placed his own hand on the tumor, which he could plainly discover, and feel the aortic pulsation quite plainly. I made the following prescription, which I directed him to use indefinitely, as I was about to leave the State; and the understanding was, that if he needed further aid he was to address me in this city. I have not heard from him, and conclude that he is improving at least. I ordered him to paint the tumor three times a day with tincture of iodine, and take the following:

| | |
|---|---|
| ℞.—Syrup Iodide Iron, . . . . . | f℥ij |
| Fowler's Solution, . . . . . . | fʒiv |
| Corrosive Sublimate, . . . . | grs. ij |
| Quinia, . . . . . . . . | grs. xx |
| Water, . . . . . . | f℥vss |

M. sig. Take one teaspoonful, three times a day, after each meal.

I will briefly review two or three cases of other forms of the disease that have fallen under my observation, and then close by referring to a case or two in which the trouble consisted principally in diagnosing *from* pancreatic disease.

In 1870 I was called in consultation in the case of a young man who was within a few days of his death from a progressively wasting disease which had existed for several months. The most prominent symptoms were those of dyspepsia, for which complaint he had been treated by several physicians until within a week or two of my call, when, owing to the extreme emaciation, a tumor was discovered in the region of his stomach. As to the nature of this tumor, and its location, there were just about as many opinions as there were doctors employed in the case: one thought it was in the stomach, another contended that it was a tumor of the liver, and a third, of very considerable experience and fair reputation, thought that it was a chronic abscess situated in some of the organs or tissues in that vicinity, and he seemed to be strengthened in this belief from the fact that there was another small tumor situated in the inguinal region. Owing to the extreme degree of emaciation of the patient, there was but little trouble in handling the tumor, which, however, was very painful under pressure; it was situated against the spinal column, roundish in shape, hard and distinctly *nodulated;* it was attended with the usual gastric and hepatic symptoms; there was an unmistakable cancerous cachexia about the appearance of the patient, besides he had lost a number of relatives with cancer and consumption. From the very deep-seated location of the tumor, it was obviously not in the liver; in the absence of any kind of a *bruit*, or decided aortic pulsation, an aneurism was out of the question. So the differentiation was evidently between scirrhus of the pylorus of the stomach and cancer of the head of the pancreas. Although there was occasional vomiting, and sometimes too of a dark-colored material, yet there was not that regular and persistent and scarcely ever-ceasing ejection of partially digested food that is always met with in cases of scirrhus and occlusion of the pylorus. I will just say here, once for all, that neither in this case, nor any other, have I had the opportunity to witness the admixture with the fæces of undigested and unemulsified fat; but I have more often met with an inordinate appetite for fatty food. This man's appetite was reduced to *nil.* This man died in about one week, and the post-mortem, at which I was not present, revealed cancer of the head of the pancreas, with a small abscess forming on the liver from mere pressure, and a small abscess in the vicinity of the cæcum.

On one occasion, seven or eight years ago, I was consulted by a strong blacksmith for what he called dyspepsia. There was uneasiness and some soreness under pressure in the region of the stomach, a good deal of trouble during digestion, but a pretty fair appetite, and a *very good* appetite for fat meat; he complained of a constant feeling of fullness in his stomach, and withal too much ill at ease to carry on his trade. On examination I found a considerable enlargement of the pancreas, which I diagnosed as chronic inflammation of the organ, and directed him to paint the skin over the tumor three times a day with tincture of iodine, and gave him ten grains of iodide of potassium three times a day. In a few days the tumor began to gradually melt away, and in three or four weeks it was entirely gone, and all the dyspeptic symptoms with it, and he went to work and has never been sick a day since to my knowledge.

In 1870 I was called in great haste to see a very strong laboring man, and found him suffering most excruciating pains in the vicinity of the pit of the stomach; it was evidently a "fit of gravel" of some kind. I at first suspected the passage of a gall-stone, but there was *no jaundice,* nor was there the nausea and vomiting of bile usually met with in such cases, and the pain was too much toward the median line of the body for a gall-stone. I diagnosed pancreotalgia from the passage of a calculus, and under the usual anodyne treatment the man was out in a day or two.

Before quitting this branch of my subject, I must make a passing remark about the purely functional troubles of this organ. Their direct diagnosis must be hypothetical; but the best way I know of to confirm the supposition is the administration of pancreatine in some form; and these are, in all probability, the cases in which the pancreatic emulsion "acts like a charm." Indeed, this remedy should be used in all cases of organic disease of the organ for the purpose of assisting digestion, until curative agents shall have had a chance to effect a cure. My experience with this remedy has not been sufficient to warrant any positive opinion as to its efficacy in such cases.

I will close this paper by giving as succinctly as possible a history of two cases of other complaints in which the diagnosis from pancreatic disease, and some other affections in that vicinity, was as difficult as possible. About a year and a half ago I was consulted by a gentleman in a neighboring county about a train of symptoms which had been treated as dyspepsia by several physicians for about two years. The patient was about fifty years of age, of rather low stature, but very corpulent, weighing then something over two hundred pounds, his healthy avoirdupois being two hundred and sixty pounds. His principal complaint was a sense of constant uneasiness in the stomach, with a variety of dyspeptic symptoms; one very prominent one I noticed was a very frequent eructation of gas. Although he is now a good citizen and prominent christian gentleman, yet I noticed from the flat appearance of his nose that the bones had been destroyed by syphilis or some other ravaging disease; and in fact, upon inquiry, he candidly informed me that in his younger days he had had tertiary syphilis and had been a hard drinker. Upon very careful examination I detected a *very deep-seated* tumor, but owing even to his present obesity, any tumor beneath the layer of adipose tissue would necessarily be deep-seated. The difficulty in locating this tumor was extreme; but in the absence of any jaundice the liver was eliminated from the count; a total absence of any *bruit* enabled me, in a measure, to exempt the pancreas and aortic aneurism. From the syphilitic taint, and from the fact that he had once been a hard drinker, I was enabled, with a pretty fair degree of certainty, to diagnose a thickening of the walls in the greater curvatuae of the stomach. I put him on the following prescription, and in two weeks he began to feel better, and in three months the tumor had almost entirely disappeared, and the dyspeptic troubles with it, and he had gained fifteen pounds in weight. I directed him to paint the skin over the tumor three times a day with the tincture of iodine, and take the following:

| | |
|---|---|
| ℞.—Iodide Potassium, . . . . . . | ʒv |
| Corrosive Sublimate, . . . . . . | grs. iij |
| Fowler's Solution, . . . . . . | ʒiv |
| Comp. sp. Lavender, . . . . . . | ℥ij |
| Water, . . . . . . . | ℥vj |

M. sig. Take one teaspoonful three times a day, after each meal. I saw this gentleman a year afterwards, and he was entirely well.

One more case, and an interesting one too, and I am done. In April, 1873, a robust young man came to consult me about a very severe pain in the region of the stomach, attended with gastric disorder and inordinate action of the heart. This pain had existed for six weeks, during which time he had lost fifteen pounds, he usually weighing two hundred and fifteen pounds. For about the four preceding years I had been treating this man for what I at first supposed was neuralgia of the head. At different times I had tried every conceivable remedy for neuralgia without ever having given him the slightest relief, except the most temporary. The pain was fixed always at one and the same spot, and some months before I came to the conclusion that there was some kind of a tumor of the brain, which would ultimately give him worse trouble. I abandoned the case and advised him to go and consult a gentleman who was one of the most accomplished physicians in Virginia; this he failed to do. When he consulted me on the present occasion, he said the "neuralgia had suddenly left his head and moved to his stomach," and such, indeed, was the case with the pain; the head was perfectly clear of pain, the first time in four years, and all of his trouble was now in the region of his stomach.

On making a most searching examination of his case, I discovered a very deep-seated tumor between the navel and pit of the stomach; it was distinctly pulsating, and expansively so, I was quite sure; there was a faint *bruit*, which, on placing him in the genu-manual position, became *more distinct*, and left no doubt on my mind but that it was an aneurismal purr. There were some dyspeptic symptoms, which never existed before; there was also a decided jaundiced hue of the eyes; also very great action of the heart, and an aneurismal murmur over the arch of the aorta. I knew his family history very well, and knew that his mother died of angina pectoris, which is but a symptom of degeneration of the coronary arteries; I knew that his sister died of *ramolissement* of the brain, which was doubtless due to thrombosis. In short, I knew that there was an hereditary predisposition to arterial degeneration. In accordance with these facts, I made the diagnosis of aneurism of the abdominal aorta, and gave a rather unfavorable opinion as to the result, which shocked him greatly. Being in preparation for a prolonged sojourn in the West, I did not undertake the treatment of his case, but sent him to the distinguished physician alluded to above.

Unfortunately this medical gentleman failed to discern the true nature of the case, but mistook it for neuralgia, and prescribed accordingly, and assured the young man that in ten days he would be so far relieved that "he would feel like another man;" and at the end of this period he should return to him, which he did, but alas! only to present himself in a condition growing progressively worse. At this visit the doctor examined him more carefully, but failed to detect the abdominal tumor, but discovered the *bruit* over the arch of the aorta, and construed it as the hygræmic murmur. This was manifestly an error, since the patient was neither, at this time, anæmic nor hygræmic, for he was very well nourished and still weighed nearly two hundred pounds. The doctor adhered to his former opinion, and noticing the jaundiced hue of the eyes and skin, and thinking that some derangement of the liver was protracting the neuralgia, he prescribed active cholagogue cathartics and applied a large blister over the epigastric region, and advised the patient to go to the warm or hot springs of Virginia. The medicine acted very drastically, and by the time he got through with it he was so much worse that he could not leave his house for some time; and he had also lost faith in the doctor, and seemed to be out of faith generally with the profession, since he had consulted two of them in whom he had confidence at least, and they differed so widely that he determined to take his case in his own hands, and did not return to his physician. But to the credit of this physician, he admitted to a friend of mine that he had been entirely mistaken in Mr. B.'s case, and that he was then satisfied that the case would terminate fatally.

Just as soon as he had sufficiently rallied from the effects of the drastics, some time towards the last of June, he went to a strong chalybeate spring, and remained there for a month or more, but on returning in July he had evidently lost ground. After having been at home for some time, the gastric and other troubles became so annoying that he was forced to call in a doctor from the neighborhood, who, with much promptness, diagnosed a case of *pure* "*dyspepsy;*" and under the use of remedies directed to the relief of the gastric symptoms, he became more comfortable for a few weeks, but he continued to lose weight. Some time in the month of October, and about five months after I had, with very great difficulty, diagnosed aortic aneurism, this doctor was called in the night to relieve some violent symptom in the case, and for the first time discovered the tumor in the patient's abdomen. The patient by this time was reduced to a mere skeleton, and long before this time the tumor must have been as plain as a big apple in an old woman's reticule. After this discovery was made, just like buzzards around carrion, a number of other doctors gathered in to render service in the case, but in spite of their combined skill the poor young man died about the first of the next January. There was no post-mortem made. Here was a case of general arterial degeneration; and as a result there were at least three aneurisms: one in the abdomen, one at the arch of the aorta, and a third located somewhere in the brain. I have introduced this case to show the great difficulty in diagnosing diseases in this region, and to show how doctors will differ as to their seat and nature.

I will now close this somewhat rambling and unphilosophical communication, in which I fear that I will be charged with prolixity, as well as tautology; but my apology is, that I am very desirous of convincing the younger members of our profession that affections of the pancreas are *by no means* uncommon, and their diagnosis and cure are by no means impossible in the majority of cases. If I succeed in this I am more than satisfied, even if I should be forced to plead guilty to the charge of inelegance of diction.—*Amer. Practitioner.*

